# Heavy metal exposures and constipation among United States adults: A cross-sectional study

**DOI:** 10.1097/MD.0000000000051070

**Published:** 2026-10-09

**Authors:** Huanhuan Lin, Shijian Wang

**Affiliations:** a Department of Gastroenterology, The Second People’s Hospital of Lianyungang & The Oncology Hospital of Lianyungang, Lianyungang, Jiangsu Province, China; b Department of Laboratory Medicine, Xuwei New District People’s Hospital, Lianyungang, Jiangsu Province, China.

**Keywords:** cadmium, constipation, heavy metals, lead, mercury, NHANES

## Abstract

Constipation is a common gastrointestinal disorder that can substantially impair quality of life. Environmental exposure to heavy metals has been implicated in gastrointestinal dysfunction; however, population-based evidence regarding their associations with constipation remains limited. This study aimed to evaluate the associations of blood lead, mercury, and cadmium levels with constipation among US adults. This cross-sectional study used data from the National Health and Nutrition Examination Survey (NHANES) 2005–2010 and included 11,014 adults aged ≥20 years. Survey-weighted multivariable logistic regression models were constructed, with metal levels analyzed as log-transformed continuous variables and quartile categories based on the original concentrations. Restricted cubic spline (RCS) analyses were performed to assess dose–response relationships. Subgroup analyses, interaction tests, and sensitivity analyses were also conducted. In the fully adjusted model, blood lead and mercury levels were each significantly inversely associated with constipation. Each 1-unit increase in log-transformed blood lead was associated with 50.4% lower odds of constipation (OR = 0.496, 95% confidence interval [CI]: 0.331–0.745), whereas each 1-unit increase in log-transformed blood mercury was associated with 29.9% lower odds (OR = 0.701, 95% CI: 0.542–0.905). Blood cadmium showed no significant association. RCS analyses further confirmed linear inverse associations of both lead and mercury with constipation, whereas no significant association was identified for cadmium. A significant interaction was identified between educational level and the association between blood lead and constipation (*P* for interaction = .04). The sensitivity analyses yielded generally consistent results. Blood lead and mercury levels were significantly and linearly inversely associated with constipation among US adults, whereas blood cadmium showed no significant association. Educational attainment may modify the association between blood lead exposure and constipation. Further prospective and mechanistic studies are needed to clarify the nature and underlying mechanisms of these associations.

## 1. Introduction

Constipation ranks as one of the most prevalent functional gastrointestinal disorders globally, significantly reducing quality of life and increasing healthcare burdens.^[1-5]^ Although dietary patterns, physical activity, and gut microbiota are closely linked to constipation,^[6-8]^ its etiology remains complex and heterogeneous, with the potential influence of environmental factors not fully elucidated. Heavy metal pollution poses a major global public health concern. Lead (Pb), mercury (Hg), and cadmium (Cd) are notable environmental heavy metals, widely distributed in the atmosphere, soil, drinking water, and food chain.^[9]^ Prolonged exposure to these metals can impair multiple organ systems through mechanisms such as oxidative stress, neurotoxicity, and gut microbiota dysbiosis.^[9-11]^ Given that the gastrointestinal tract is a primary site for exposure to and absorption of exogenous substances, it may be particularly susceptible to the toxic effects of heavy metals.

Previous studies indicate that heavy metal exposure may influence bowel function through various biological pathways.^[9,10,12]^ Nonetheless, direct epidemiological evidence linking heavy metal exposure to bowel function in the general population remains scarce. Investigations based on the National Health and Nutrition Examination Survey (NHANES) have suggested that elevated blood lead levels may correlate with lower odds of constipation.^[13,14]^ Additionally, Xie and Lei noted a potential inverse relationship between blood mercury levels and constipation.^[14]^ However, prior studies have primarily concentrated on the association between blood lead and constipation,^[13]^ or examined multiple metals without systematically comparing the association patterns of blood lead, mercury, and cadmium for constipation within the same multivariable model.^[14]^ Consequently, the relationship between heavy metal exposure and constipation is not fully understood. Furthermore, while Li et al conducted subgroup and interaction analyses involving socioeconomic factors such as educational attainment, no significant modifying effect was observed.^[13]^ Whether the association between heavy metal exposure and constipation varies across differing socioeconomic backgrounds merits further investigation.

Thus, using NHANES 2005–2010 data, this study concurrently evaluated the co-exposure to blood lead, mercury, and cadmium to systematically assess their associations with constipation. Additionally, dose–response relationships and potential effect modifiers were analyzed to enhance understanding of the links between low-level environmental heavy metal exposure and constipation.

## 2. Materials and methods

### 2.1. Data source and study population

This study utilized data from 3 cycles of the NHANES: 2005–2006, 2007–2008, and 2009–2010. NHANES, a nationally representative survey conducted by the National Center for Health Statistics (NCHS), employs a complex multistage probability sampling design. The NHANES protocol received approval from the NCHS Research Ethics Review Board, with all participants providing written informed consent. In accordance with the Centers for Disease Control and Prevention (CDC) analytic guidelines, all analyses incorporated NHANES sampling weights, stratification, and clustering design variables, alongside Mobile Examination Center (MEC) examination weights (WTMEC2YR).^[15]^

Eligibility criteria for participants included being adults aged ≥20 years, having complete measurements of blood lead (LBXBPB), blood cadmium (LBXBCD), and blood mercury (LBXTHG), as well as complete bowel habit information.

Exclusions were made for pregnant individuals, those with colorectal cancer, individuals with missing or implausible values for key variables (e.g., extreme dietary energy intake), or those lacking estimated glomerular filtration rate (eGFR) values. The eGFR was determined using the 2021 Chronic Kidney Disease Epidemiology Collaboration (CKD-EPI) creatinine equation.

### 2.2. Variable definitions

#### 2.2.1. Exposure variables

According to the NHANES laboratory protocol, blood concentrations of Pb, Cd, and total Hg were measured using inductively coupled plasma mass spectrometry (ICP-MS). Values below the limit of detection (LOD) were substituted with LOD/√2.^[16]^ Concentrations of all 3 blood heavy metals were expressed in μg/L, with blood lead concentrations converted from μg/dL to μg/L for consistency.

#### 2.2.2. Outcome variable

Bowel habits were assessed using the Bowel Health Questionnaire (BHQ).^[17]^ Participants were classified as having constipation if they met either criterion: a bowel movement frequency of <3 times per week or a stool consistency classified as Bristol Stool Form Scale (BSFS) type 1 or 2; types 3 to 7 were categorized as non-constipation.^[13,18]^

#### 2.2.3. Covariates

Based on previous literature and clinical relevance, the analyses included the following covariates: age (20–39 years, 40–59 years, and ≥60 years), sex, race/ethnicity (Mexican American, non-Hispanic White, non-Hispanic Black, other Hispanic, and other races), educational level (<9 years, 9–11 years, high school graduate (General Educational Development) GED or equivalent, college or associate degree, and college graduate or above), family poverty income ratio (PIR; ≤1.30, 1.31–3.49, and ≥3.50), marital status (unmarried, married/living with partner, widowed/divorced/separated), smoking status (current/former/never), physical activity level (moderate and vigorous physical activity), hypertension, diabetes mellitus (participants with prediabetes were classified into the non-diabetes group), eGFR (treated as a continuous variable in primary analyses), and body mass index (BMI; <25, 25–29.99, and ≥30 kg/m^2^).

Dietary data were collected via two 24-hour dietary recall interviews. In instances where both recalls were available, their mean value was utilized; otherwise, only the first-day recall was analyzed. Dietary variables encompassed total energy intake, water intake, caffeine intake, alcohol intake, dietary supplement use, and dietary fiber density (g/1000 kcal). Alcohol intake was categorized as nondrinker, mild (≤14 g/day for women, ≤28 g/day for men), or excessive (>14 g/day for women, >28 g/day for men).

### 2.3. Statistical analysis

All statistical analyses accounted for the complex sampling design of the NHANES, including stratification (SDMVSTRA), clustering (SDMVPSU), and sample weights. In accordance with NHANES analytical guidelines, the 2-year MEC examination weights (WTMEC2YR) from the 3 survey cycles (2005–2010) were divided by 3 to create combined sample weights.

Continuous variables were presented as weighted medians with interquartile ranges (IQRs), and group differences were assessed using the weighted Mann–Whitney *U* test. Categorical variables were expressed as weighted percentages and compared via the Rao–Scott χ^2^ test. Given the right-skewed distributions of blood metal concentrations, logarithmic transformation was applied to mitigate the impact of extreme values and enhance model stability.

Survey-weighted correlation coefficients were computed to evaluate correlations among blood lead, cadmium, and mercury levels, with results visualized in a heatmap.

Survey-weighted multivariable logistic regression models were developed to explore the associations between blood heavy metal levels and constipation, estimating odds ratios (ORs) and 95% confidence intervals (CIs). Blood levels of lead, cadmium, and mercury were analyzed both as log-transformed continuous variables and as categorical variables based on quartiles, with the lowest quartile (Q1) designated as the reference group.

Three multivariable logistic regression models were formulated as follows: Model 1 included mutual adjustment for the 3 blood metals; model 2 incorporated additional adjustments for age, sex, race/ethnicity, educational level, marital status, and PIR; model 3 further adjusted for BMI, smoking status, alcohol intake, hypertension, diabetes, dietary energy intake, dietary fiber density, water intake, caffeine intake, dietary supplement use, physical activity, and eGFR. Linear trends across quartiles were assessed by treating quartile categories as ordinal variables within the models.

Variance inflation factors (VIFs) were calculated to evaluate potential multicollinearity among covariates. To assess possible nonlinear associations, restricted cubic spline (RCS) models with 4 knots were fitted within the fully adjusted model 3 survey-weighted logistic regression framework using log-transformed continuous metal concentrations. The knots were positioned at the fifth, 35th, 65th, and 95th percentiles of the exposure distribution, with the analysis limited to the fifth to 95th percentiles to reduce the influence of extreme values. The median concentration of each metal served as the reference point. Wald tests were conducted to evaluate overall and nonlinear associations.

Subgroup analyses were executed using survey-weighted multivariable logistic regression models. Interaction terms between each metal and subgroup variables were introduced into the fully adjusted model, with significance assessed using Wald tests at a threshold of *P* < .05.

Sensitivity analyses were performed based on the fully adjusted model 3 to evaluate the robustness of findings, including models without adjustment for eGFR and analyses excluding extreme heavy metal values (defined as values below the first percentile or above the 99th percentile), the latter being restricted to continuous exposure models.

All statistical analyses employed R software (version 4.5.3; R Foundation for Statistical Computing, Vienna, Austria), utilizing key R packages including the survey package for complex sampling analysis, rms for RCS modeling, and ggplot2 for visualization. A 2-sided *P* value < .05 was deemed statistically significant.

Statistical analyses followed established NHANES analytic guidelines for complex survey data, with analytical procedures independently performed and verified by authors experienced in epidemiological analysis of population-based datasets.

This study adhered to the Strengthening the Reporting of Observational Studies in Epidemiology (STROBE) guidelines for cross-sectional studies.

## 3. Results

A total of 31,034 participants from NHANES 2005–2010 were initially included in the analysis. After excluding individuals aged <20 years (n = 13,902), those with missing constipation-related data (n = 2385), and those lacking blood heavy metal data (n = 553), 14,194 participants remained. Further exclusions included pregnant women (n = 395) and participants with colorectal cancer (n = 1333), resulting in 12,466 eligible participants.

Subsequently, participants with missing covariates were excluded, including those with incomplete educational level (n = 14), marital status (n = 5), PIR (n = 890), smoking status (n = 4), hypertension data (n = 19), diabetes data (n = 8), BMI (n = 102), first-day dietary recall data (n = 171), implausible energy intake (n = 120; defined as <500 or >8000 kcal/day for men and <500 or >5000 kcal/d for women), and missing dietary supplement information (n = 3). Additionally, participants with missing eGFR data (n = 116) were excluded. Ultimately, 11,014 participants were included in the final analysis (Fig. 1).

**Figure 1.**
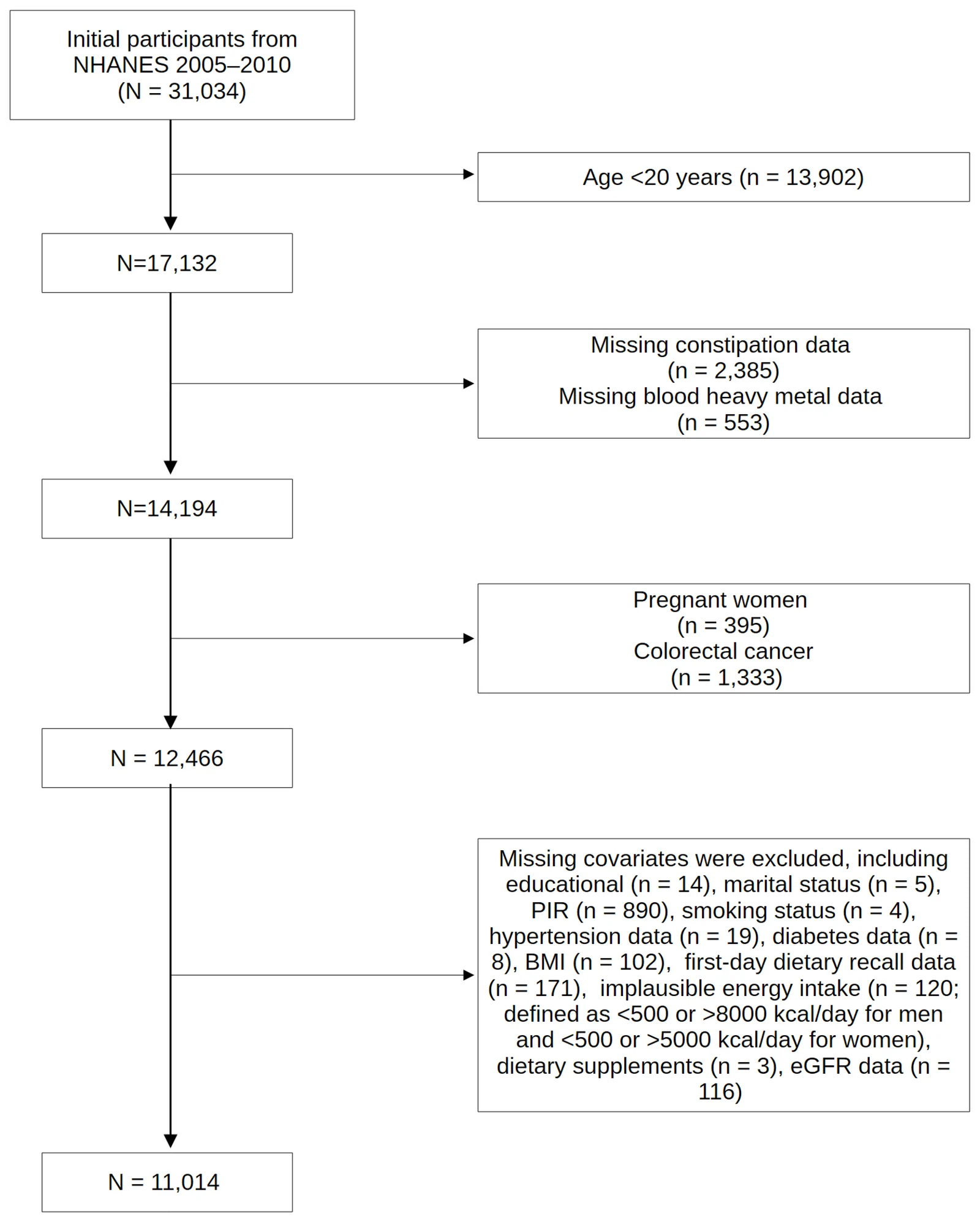
Flowchart of participant selection. BMI = body mass index, eGFR = estimated glomerular filtration rate, NHANES = National Health and Nutrition Examination Survey, PIR = poverty–income ratio.

### 3.1. Baseline characteristics of participants

The final study population of 11,014 participants represents approximately 152 million US adults.

When comparing participants with constipation to those without, individuals with constipation were more likely to be younger (20–39 years), female, non-Hispanic Black, physically inactive (lacking moderate or vigorous physical activity), possess lower educational attainment (high school or below), and have lower PIRs (all *P* < .05). Significant differences between the 2 groups were also noted concerning marital status, BMI, smoking status, alcohol intake, and dietary factors, including energy intake, dietary fiber density, water intake, and caffeine intake (all *P* < .05).

Regarding heavy metal biomarkers, participants with constipation exhibited significantly lower blood lead and mercury levels than those without constipation (both *P* < .001); however, no significant difference in blood cadmium levels was found between the 2 groups.

No significant differences were detected in the prevalence of hypertension or diabetes. Although categorized eGFR stages did not significantly differ between groups, participants with constipation presented slightly higher continuous eGFR levels than those without constipation (100.65 vs 97.90 mL/min/1.73 m^2^, *P* = .003).

Detailed results are outlined in Table 1.

**Table 1:** Baseline characteristics of study participants according to constipation status.

Variable	Overall (N = 11,014)	No constipation (n = 9939)	Constipation (n = 1075)	*P*
Age (%)				.004
20–39	3880 (38.8)	3434 (38.2)	446 (44.7)	
40–59	3853 (40.9)	3487 (41.2)	366 (38.2)	
≥60	3281 (20.3)	3018 (20.6)	263 (17.1)	
Gender (%)				<.001
Female	5355 (49.5)	4613 (47.1)	742 (73.5)	
Male	5659 (50.5)	5326 (52.9)	333 (26.5)	
Race (%)				<.001
Mexican American	2073 (8.3)	1883 (8.3)	190 (8.7)	
NH Black	2179 (10.7)	1895 (10.1)	284 (16.5)	
NH White	5406 (71.4)	4941 (72.0)	465 (65.5)	
Other Hispanic	901 (4.2)	801 (4.2)	100 (4.9)	
Other race	455 (5.3)	419 (5.4)	36 (4.4)	
Education (%)				<.001
Less than ninth grade	1230 (5.6)	1098 (5.4)	132 (6.7)	
9–11th grade	1792 (12.3)	1581 (12.0)	211 (15.3)	
High school graduate/GED or equivalent	2627 (24.1)	2321 (23.5)	306 (29.9)	
Some college or AA degree	3120 (30.9)	2846 (31.2)	274 (28.5)	
College graduate or above	2245 (27.1)	2093 (27.8)	152 (19.4)	
Marital status (%)				<.001
Married	6780 (65.3)	6183 (65.9)	597 (59.2)	
Sep/div/wid	2371 (17.5)	2111 (17.1)	260 (21.6)	
Unmarried	1863 (17.1)	1645 (16.9)	218 (19.3)	
PIR (%)				<.001
≤1.30	3279 (19.1)	2860 (18.2)	419 (28.0)	
1.31–3.49	3559 (45.2)	3310 (35.5)	249 (38.1)	
≥3.50	4176 (35.7)	3769 (46.3)	407 (34.0)	
BMI (%)				<.001
<25	3137 (31.1)	2761 (30.4)	376 (38.4)	
25–29.9	4097 (35.2)	3746 (33.9)	351 (32.4)	
≥30	3780 (33.7)	3432 (35.7)	348 (29.2)	
Smoking (%)				<.001
Current	2538 (22.9)	2287 (22.8)	251 (24.3)	
Former	2699 (24.1)	2503 (24.7)	196 (17.1)	
Never	5777 (53.0)	5149 (52.5)	628 (58.5)	
Alcohol (%)				<.001
Excessive	1652 (17.6)	1542 (18.1)	110 (11.9)	
Mild	1927 (19.3)	1791 (19.9)	136 (13.5)	
Nondrinker	7435 (63.2)	6606 (62.0)	829 (74.7)	
Moderate activity (%)				<.001
No	3068 (22.5)	2707 (22.0)	361 (28.0)	
Yes	7946 (77.5)	7232 (78.0)	714 (72.0)	
Vigorous activity (%)				.01
No	7158 (60.1)	6416 (59.6)	742 (65.4)	
Yes	3856 (39.9)	3523 (40.4)	333 (34.6)	
Hypertension (%)				.06
No	7396 (71.2)	6624 (71.0)	772 (74.0)	
Yes	3618 (28.8)	3315 (29.0)	303 (26.0)	
Diabetes (%)				.64
No	9837 (92.5)	8881 (92.5)	956 (92.1)	
Yes	1177 (7.5)	1058 (7.5)	119 (7.9)	
eGFR stage (%)				.08
<60	766 (4.7)	681 (4.6)	85 (5.6)	
60–89	3436 (30.6)	3145 (30.9)	291 (27.5)	
≥90	6812 (64.7)	6113 (64.5)	699 (66.9)	
Energy (%)				<.001
T1	3674 (29.4)	3217 (28.4)	457 (39.8)	
T2	3671 (33.7)	3326 (33.8)	345 (32.7)	
T3	3669 (36.9)	3396 (37.9)	273 (27.5)	
Fiber density (%)				<.001
T1	3672 (35.0)	3243 (34.3)	429 (42.7)	
T2	3671 (34.1)	3334 (34.3)	337 (32.0)	
T3	3671 (30.8)	3362 (31.4)	309 (25.3)	
Water intake (%)				<.001
T1	3672 (26.5)	3161 (24.9)	511 (42.8)	
T2	3671 (33.9)	3341 (34.0)	330 (33.2)	
T3	3671 (39.5)	3437 (41.1)	234 (24.0)	
Caffeine (%)				<.001
T1	3679 (28.1)	3240 (27.3)	439 (36.2)	
T2	3669 (31.8)	3311 (31.8)	358 (31.8)	
T3	3666 (40.1)	3388 (40.9)	278 (32.0)	
Supplement (%)				<.001
No	5827 (49.3)	5193 (48.5)	634 (56.6)	
Yes	5187 (50.7)	4746 (51.5)	441 (43.4)	
Lead (%)				<.001
Q1	2754 (28.4)	2380 (27.2)	374 (41.3)	
Q2	2757 (26.7)	2495 (26.9)	262 (25.2)	
Q3	2767 (24.0)	2549 (24.6)	218 (17.5)	
Q4	2736 (20.8)	2515 (21.3)	221 (16.0)	
Cadmium (%)				.17
Q1	3013 (30.3)	2701 (30.2)	312 (31.1)	
Q2	2643 (24.6)	2414 (24.9)	229 (21.5)	
Q3	2604 (21.5)	2348 (21.5)	256 (21.1)	
Q4	2754 (23.6)	2476 (23.3)	278 (26.2)	
Mercury (%)				<.001
Q1	2816 (23.6)	2464 (22.8)	352 (32.4)	
Q2	2731 (23.8)	2460 (23.8)	271 (24.3)	
Q3	2724 (24.9)	2469 (24.9)	255 (24.5)	
Q4	2743 (27.6)	2546 (28.5)	197 (18.7)	
eGFR (mL/min/1.73 m^2^) (median [IQR])	98.18 [82.93, 111.15]	97.90 [82.90, 110.67]	100.65 [83.89, 114.96]	.003
log lead (µg/L) (median [IQR])	1.11 [0.92, 1.30]	1.11 [0.93, 1.30]	1.02 [0.86,1.24]	<.001
log cadmium (µg/L) (median [IQR])	−0.51 [−0.70, −0.24]	−0.51 [−0.70, −0.24]	−0.48 [−0.70, −0.20]	.14
log mercury (µg/L) (median [IQR])	-0.01 [-0.28, 0.28]	0.00 [−0.27, 0.29]	−0.10 [−0.39, 0.15]	<.001

The values are presented as unweighted counts (n) and weighted percentages (%) or weighted medians (IQRs), as appropriate. *P* values were calculated using survey-weighted methods accounting for the complex sampling design.

Quartiles of blood heavy metals (μg/L): Blood lead: Q1 (1.8–8.92), Q2 (8.92–13.9), Q3 (13.9–21.6), and Q4 (21.6–331); blood mercury: Q1 (0.14–0.50), Q2 (0.50–0.91), Q3 (0.91–1.73), and Q4 (1.73–85.7); blood cadmium: Q1 (0.14–0.22), Q2 (0.22–0.34), Q3 (0.34–0.608), and Q4 (0.608–10.8).

BMI = body mass index, eGFR = estimated glomerular filtration, GED = General Educational Development, NH = Non-Hispanic, PIR = poverty income ratio, IQR = interquartile range.

### 3.2. Correlation analysis of blood heavy metals

Survey-weighted correlation analysis revealed a moderate positive correlation between blood lead and cadmium levels (*r* = 0.33, *P* < .001), while correlations between lead and mercury (*r* = 0.11, *P* < .001) and between cadmium and mercury (*r* = −0.07, *P* < .001) were weak. All correlation coefficients were below 0.4, indicating no evidence of strong correlations among the blood metals, thus supporting their simultaneous inclusion in regression models (Fig. 2).

**Figure 2.**
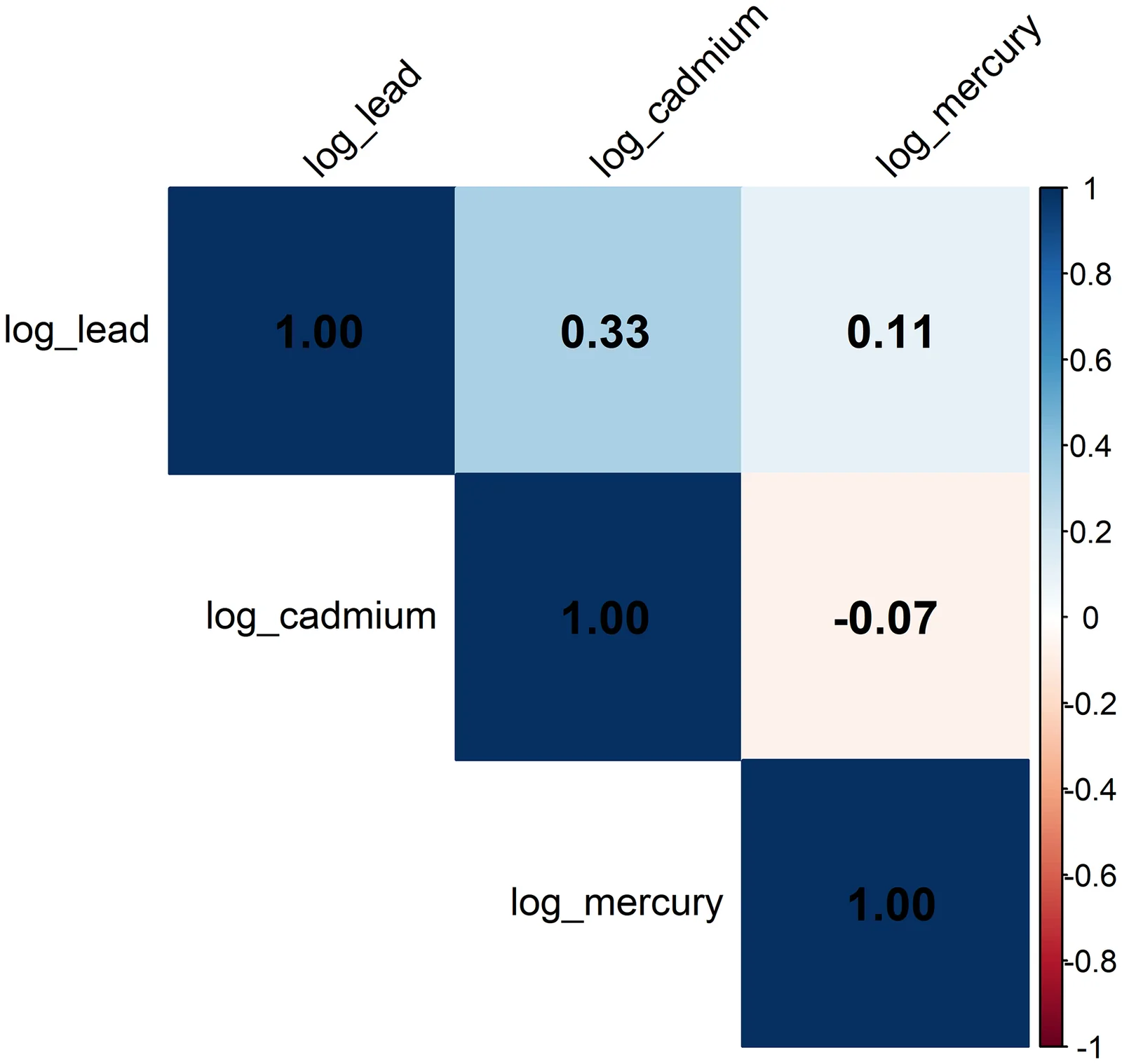
Survey-weighted correlation matrix of blood lead, cadmium, and mercury levels.

### 3.3. Associations between blood heavy metals and constipation

In analyses utilizing continuous variables, blood lead and mercury levels demonstrated inverse associations with constipation in the fully adjusted model (model 3). Specifically, each 1-unit increase in log-transformed blood lead levels correlated with a 50.4% reduction in the odds of constipation (OR = 0.496, 95% CI: 0.331–0.745), while each 1-unit increase in log-transformed blood mercury levels was associated with a 29.9% reduction in the odds of constipation (OR = 0.701, 95% CI: 0.542–0.905). No significant association was found for cadmium.

In quartile-based analyses, ORs for all higher exposure categories of blood lead and mercury, compared to the lowest quartile (Q1), were below 1.0 with relatively narrow CIs, indicating overall inverse associations with constipation, although not all comparisons reached statistical significance. While point estimates for all cadmium quartiles were below 1.0, CIs were wide and crossed 1.0, suggesting instability in the association between blood cadmium levels and constipation.

Significant dose–response trends were evident for both lead (*P* for trend = .04) and mercury (*P* for trend = .03), whereas no significant trend was observed for cadmium.

The forest plots from model 3 are depicted in Figure 3A, B.

**Figure 3.**
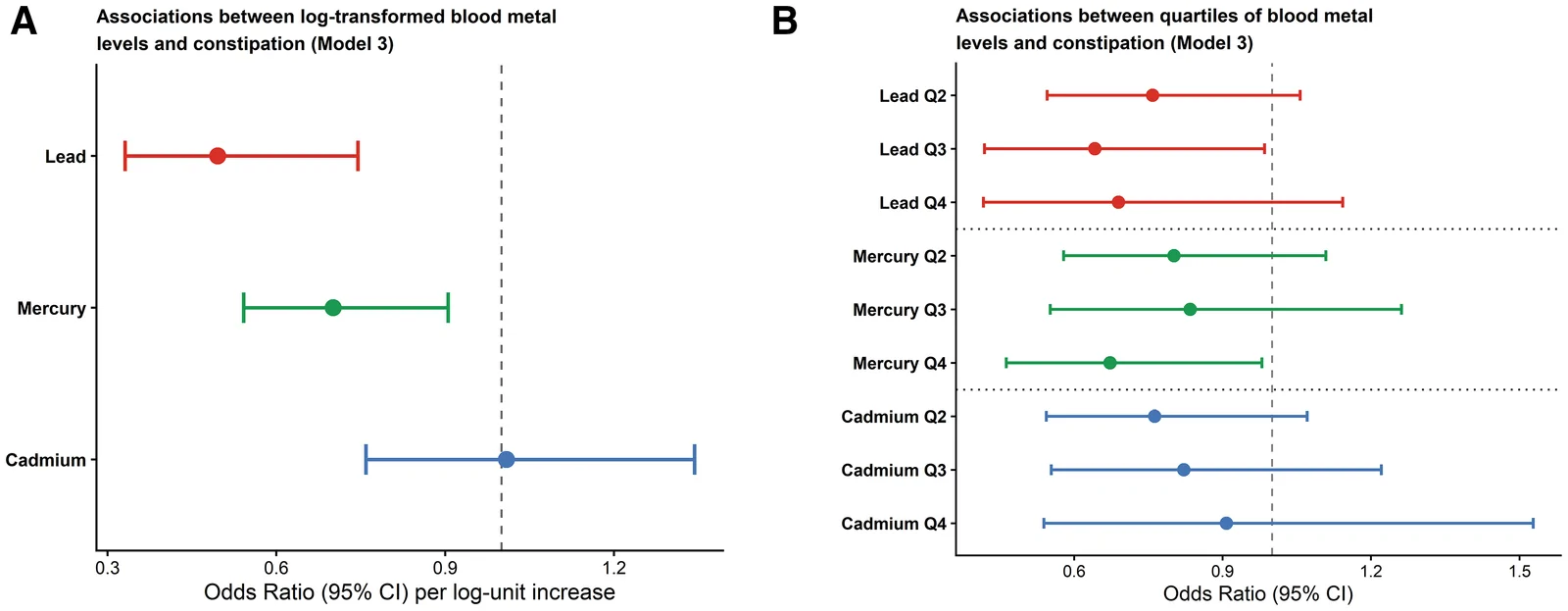
Associations between blood heavy metals and constipation. (A) Continuous variables (log-transformed). (B) Quartiles. CI = confidence interval.

Complete regression results for all models, including ORs, 95% CIs, and *P* values, are detailed in Supplemental Digital Content S1, Supplemental Digital Content 1, which presents the full survey-weighted logistic regression analyses of blood heavy metals and constipation.

### 3.4. Dose–response relationships between blood heavy metals and constipation

Following adjustments for all potential confounders and survey weights, RCS analyses were conducted to evaluate dose–response relationships. Blood lead levels exhibited a significant inverse association with constipation (overall *P* < .001), with no evidence of nonlinearity, indicating an approximately linear relationship. Likewise, blood mercury levels significantly inversely correlated with constipation (overall *P* < .001), with no significant nonlinear relationship observed. Conversely, no significant association was identified between blood cadmium levels and constipation.

VIF analyses indicated that all covariates had VIF values below 2 (range: 1.04–1.46), suggesting no substantial multicollinearity.

The results of the RCS analyses are illustrated in Figure 4A, C.

**Figure 4.**
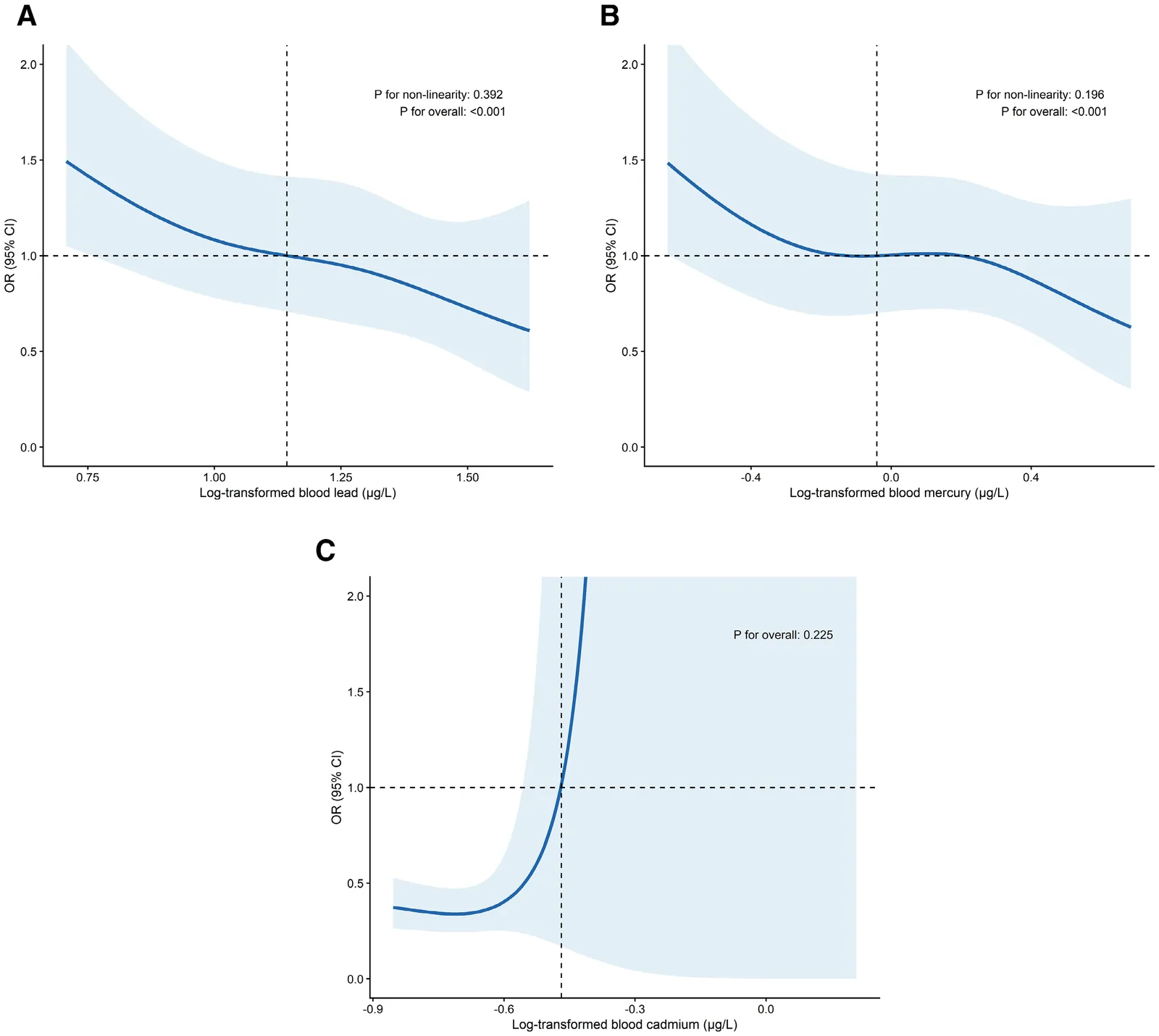
Restricted cubic spline analyses of associations between blood heavy metal levels and constipation. (A) Blood lead. (B) Blood mercury. (C) Blood cadmium. The solid lines represent adjusted odds ratios (ORs), and the shaded areas represent 95% confidence intervals (CIs). The horizontal dashed line indicates OR = 1.0, and the vertical dashed line indicates the reference concentration (median concentration of each metal). All models were adjusted for age, sex, race/ethnicity, educational level, marital status, PIR, BMI, smoking status, alcohol intake, hypertension, diabetes, energy intake, dietary fiber density, water intake, caffeine intake, dietary supplement use, physical activity, eGFR, and the other 2 blood metals. BMI = body mass index, CI = confidence interval, eGFR = estimated glomerular filtration rate, PIR = poverty–income ratio, OR = odds ratio.

### 3.5. Subgroup and interaction analyses

In subgroup analyses, the inverse association between blood lead levels and constipation was generally consistent across most subgroups, with interaction analyses revealing a significant modifying effect of educational level on this association (*P* for interaction = .04). Stratified analyses indicated that the inverse relationship between blood lead levels and constipation was more pronounced among participants with higher educational attainment. No significant interactions were noted for the remaining covariates.

For blood mercury levels, the inverse association with constipation remained generally stable across subgroups. Although relatively stronger associations were observed in specific subgroups, such as women and never smokers, no statistically significant interactions were detected (all *P* values for interactions > .05).

For blood cadmium levels, statistically significant associations were seen in only a few subgroups, while no significant interaction effects were identified overall.

The subgroup and interaction analysis results are presented in Figure 5A, C, with complete stratified and interaction estimates available in Supplemental Digital Contents S2–S4, Supplemental Digital Content 2.

**Figure 5.**
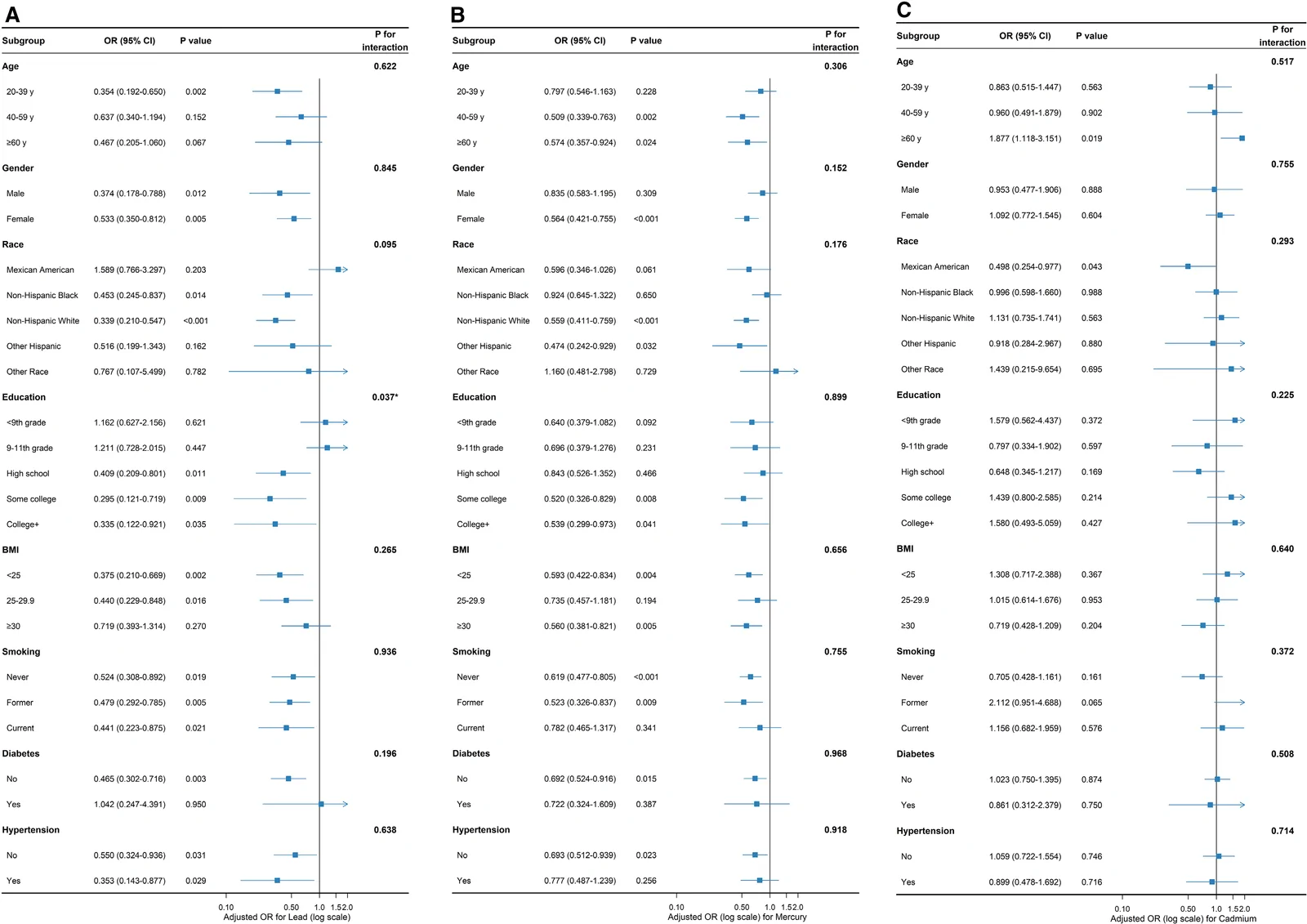
Subgroup and interaction analyses of associations between blood heavy metals and constipation. (A) Blood lead. (B) Blood mercury. (C) Blood cadmium. Adjusted for all covariates included in model 3 except the stratification variable itself. BMI = body mass index, CI = confidence interval, OR = odds ratio.

### 3.6. Sensitivity analyses

Sensitivity analyses yielded results consistent with the primary analyses. The inverse associations of blood lead and mercury with constipation remained statistically significant after excluding adjustments for eGFR and after removing extreme exposure values, while blood cadmium continued to show no significant association with constipation.

Detailed results are provided in Table 2.

**Table 2:** Sensitivity analyses: associations between blood heavy metal levels and constipation.

Exposure	Primary analysis (model 3) OR (95% CI)	*P*	Sensitivity 1: Excluding eGFR OR (95% CI)	*P*	Sensitivity 2: Excluding extreme values OR (95% CI)	*P*
Continuous variables (log-transformed)						
Lead	0.496 (0.331–0.745)	.004	0.502 (0.336–0.750)	.003	0.459 (0.248–0.850)	.02
Cadmium	1.009 (0.759–1.343)	.94	1.013 (0.768–1.335)	.92	1.091 (0.653–1.824)	.71
Mercury	0.701 (0.542–0.905)	.01	0.700 (0.544–0.900)	.01	0.752 (0.583–0.969)	.03
Categorical variables (quartiles)						
Lead Q1	Reference	–	Reference	–	–	–
Lead Q2	0.759 (0.546–1.057)	.08	0.761 (0.571–1.015)	.06	–	–
Lead Q3	0.642 (0.419–0.985)	.05	0.646 (0.443–0.941)	.03	–	–
Lead Q4	0.690 (0.417–1.143)	.10	0.695 (0.449–1.077)	.08	–	–
Cadmium Q1	Reference	–	Reference	–	–	–
Cadmium Q2	0.763 (0.544–1.071)	.09	0.765 (0.571–1.024)	.06	–	–
Cadmium Q3	0.822 (0.554–1.221)	.21	0.824 (0.584–1.163)	.19	–	–
Cadmium Q4	0.908 (0.539–1.528)	.60	0.910 (0.581–1.425)	.59	–	–
Mercury Q1	Reference	–	Reference	–	–	–
Mercury Q2	0.802 (0.579–1.109)	.12	0.801 (0.604–1.064)	.10	–	–
Mercury Q3	0.835 (0.552–1.262)	.26	0.834 (0.582–1.195)	.23	–	–
Mercury Q4	0.673 (0.463–0.980)	.04	0.673 (0.485–0.933)	.03	–	–

The primary analysis was fully adjusted as in model 3.

Sensitivity analysis 1 excluded adjustment for eGFR.

Sensitivity analysis 2 excluded extreme metal values (below the first percentile or above the 99th percentile) and was performed only for continuous exposure models.

ORs and 95% CIs were estimated using survey-weighted logistic regression models.

CI = confidence interval, eGFR = estimated glomerular filtration rate, OR = odds ratio.

## 4. Discussion

In this study, 11,014 nationally representative US adults from the NHANES 2005–2010 cycles were analyzed to systematically evaluate the associations between blood lead, cadmium, and mercury levels and constipation. Using survey-weighted multivariable logistic regression, RCS analyses, subgroup and interaction analyses, and sensitivity analyses, the relationships between heavy metal exposure and constipation were comprehensively assessed.

Key findings included significant associations between higher blood lead and mercury levels and lower odds of constipation, with RCS analyses supporting linear dose–response relationships. Conversely, no significant association was found between blood cadmium levels and constipation. These results remained consistent in sensitivity analyses excluding extreme heavy metal values and omitting adjustments for eGFR. Importantly, a significant interaction between educational attainment and blood lead levels concerning constipation was noted (*P* for interaction = .04). This study provides additional population-based evidence regarding the association between environmental heavy metal exposure and constipation by simultaneously considering co-exposure to multiple heavy metals and adjusting for a wide range of dietary, socioeconomic, and clinical factors.

Previous NHANES-based studies have reported inverse associations between blood lead or mercury levels and constipation, aligning with our findings. Xie and Lei first documented a negative association between blood lead levels and constipation in a preprint study. Notably, while the association between blood mercury and constipation was not statistically significant in their generalized linear model, a significant inverse relationship was observed using weighted quantile sum (WQS) mixture models.^[14]^ Subsequently, Li et al reported an inverse association between blood lead levels and constipation in a fully adjusted model.^[13]^ Both studies utilized RCS analyses, revealing significant nonlinear dose–response relationships between blood lead and constipation (nonlinearity *P* < .01), while evidence for the dose–response relationship between blood mercury and constipation remains limited. Our findings, showing no significant association between blood cadmium levels and constipation, are consistent with previous NHANES studies.^[14]^

The present study differs from prior research in several methodological aspects. First, blood lead, mercury, and cadmium levels were concurrently included in the same regression model, allowing for comparison of association patterns among different heavy metals while accounting for co-exposure. Second, unlike earlier studies that reported nonlinear dose–response relationships based on raw exposure values, our analysis identified more stable linear inverse associations between blood lead, mercury, and constipation after logarithmic transformation of metal concentrations. This difference may reflect variations in exposure transformation. Additionally, the study adjusted for several more covariates, including eGFR and dietary supplement use, and excluded participants with colorectal cancer, which may reduce residual confounding.

Previous studies have indicated that acute high-level lead exposure is often associated with abdominal colic and constipation.^[19]^ However, the median blood lead concentration in the present study (13.9 μg/L) falls within the typical range observed in the general population, suggesting that the findings may primarily reflect the association between chronic low-level lead exposure and constipation. Several mechanisms may help explain this association. First, lead exposure is known to induce oxidative stress,^[20-23]^ which may contribute to enteric neuronal dysfunction.^[24,25]^ Additionally, lead exposure has been linked to central nervous system injury and autonomic dysfunction.^[9,11,21,26,27]^ Both autonomic dysregulation and enteric nervous system impairment have been associated with altered gastrointestinal motility and bowel habits,^[28,29]^ while central nervous system dysfunction may further influence intestinal motility through the gut–brain axis.^[30]^ Thus, chronic low-level lead exposure may affect bowel habits via mechanisms related to neural regulation of gastrointestinal function. Second, lead exposure has been reported to alter gut microbial composition,^[10,31-34]^ with gut microbiota and its metabolites influencing intestinal motility and stool characteristics.^[35,36]^ These alterations may also contribute to the observed inverse association with constipation. Furthermore, lead exposure may impair intestinal mucosal barrier integrity and increase intestinal permeability by reducing the expression of tight junction proteins such as ZO-1 and occludin.^[34,37]^ Disruption of intestinal barrier function could additionally affect intestinal physiology and bowel function,^[38,39]^ potentially contributing to the decreased risk of constipation. Lead exposure has also been associated with alterations in thyroid function,^[23,40]^ providing another potential pathway linking lead exposure to bowel habits.^[13]^ Nevertheless, direct evidence supporting these mechanisms is limited, and residual confounding factors should be considered. Despite adjustments for a wide range of covariates, unmeasured environmental or lifestyle factors may still influence the observed associations.

Notably, a significant interaction was found between educational attainment and the association between blood lead levels and constipation (*P* for interaction = .04). Stratified analyses indicated that the inverse association was more pronounced among participants with higher educational attainment. To our knowledge, this finding has not been reported in NHANES-based studies.^[13]^ The interaction remained significant after further adjustments for dietary fiber intake and water consumption, implying that socioeconomic status (SES), as reflected by educational attainment, may influence the association between lead exposure and constipation through mechanisms beyond dietary factors. Previous studies have suggested that SES correlates with chronic stress levels,^[41-43]^ which may, in turn, influence gut microbial composition.^[44]^ Given the potential role of gut microbiota and its metabolites in gastrointestinal motility,^[35,36]^ these pathways may provide a possible explanation for the observed interaction with educational attainment. However, direct evidence to substantiate this hypothesis is still limited. Additionally, residual confounding from unmeasured factors, such as early-life exposures, specific dietary patterns, or micronutrient intake, cannot be entirely ruled out. Future prospective studies are needed to determine whether the observed heterogeneity reflects true effect modification or residual confounding.

Similar to lead exposure, mercury exposure may also influence bowel habits and gastrointestinal function through mechanisms such as neurotoxicity, oxidative stress, gut microbiota dysbiosis, and impairment of the intestinal mucosal barrier.^[45-48]^ These mechanisms may help explain the inverse association observed between blood mercury levels and constipation in this study. Given that these pathways have been discussed previously, they will not be reiterated here.

Moreover, unlike lead, blood mercury levels in the general US population primarily reflect methylmercury exposure derived from fish and seafood consumption.^[49,50]^ Previous research has indicated that dietary intake of seafood-derived docosahexaenoic acid (DHA) and eicosapentaenoic acid (EPA) may be associated with a reduced risk of constipation.^[51]^ However, the prior study^[51]^ did not include measurements of blood mercury, and the present study did not evaluate source-specific n-3 fatty acid intake. Seafood serves as both the primary source of methylmercury exposure and a significant dietary source of n-3 polyunsaturated fatty acids, such as DHA and EPA. Given that these exposures often coexist within the population, disentangling their independent contributions to constipation risk in epidemiological studies can be challenging. Thus, the inverse association noted in the present study may be influenced, at least in part, by residual confounding related to seafood-associated dietary patterns.

Notably, previous studies have demonstrated that cadmium exposure can induce oxidative stress, neurotoxicity, gut microbiota dysbiosis, and impairment of intestinal barrier function.^[9,46,47,52-54]^ However, no significant association between blood cadmium levels and constipation was observed in the present study. The mechanisms underlying this discrepancy remain unclear. Possible explanations may include differences in tissue distribution, primary target organs, and toxicological manifestations among various heavy metals,^[9]^ as well as limited statistical power and residual confounding. Additionally, the study population was generally exposed to relatively low levels of cadmium, indicating that our findings predominantly reflect associations under conditions of low-level environmental exposure. Further investigation is warranted to assess whether different effects may emerge at higher levels of cadmium exposure. These findings suggest that additional studies are necessary to better elucidate the relationships between different heavy metals and constipation.

This study possesses several strengths. First, the analysis utilized the nationally representative NHANES database and incorporated complex survey weighting, enhancing the generalizability of the findings to the US adult population. Second, multiple blood heavy metals were included simultaneously in the models, allowing for consideration of potential co-exposure effects. Additionally, a broad range of covariates, including dietary and physiological factors, was adjusted for, which improved the robustness of the analyses. Finally, consistency observed across multiple sensitivity analyses further strengthened the reliability of the findings.

However, several limitations should be acknowledged. First, due to the cross-sectional design, causal relationships cannot be established. Furthermore, single blood measurements may not accurately reflect long-term exposure levels. Second, constipation was defined based on self-reported questionnaire data, potentially introducing recall bias. Finally, although extensive adjustments were made, residual confounding from dietary factors and other unmeasured variables cannot be entirely excluded.

## 5. Conclusion

In conclusion, utilizing nationally representative NHANES data, this study found that blood lead and mercury levels exhibited significant linear inverse associations with constipation prevalence among US adults, while no similar association was found for blood cadmium. By considering multimetal co-exposure and adjusting for a wide range of potential confounders, this study highlights the complexity of associations between low-level environmental heavy metal exposure and constipation. Importantly, educational level significantly modified the association between blood lead levels and constipation, suggesting that socioeconomic factors may influence the relationship between environmental exposure and constipation. Further prospective studies and mechanistic investigations are needed to clarify the nature and underlying mechanisms of these associations.

## Acknowledgments

During manuscript preparation, AI-assisted tools were used for English language translation and assistance with R code writing and debugging. The authors independently performed, verified, and interpreted all analyses and take full responsibility for the study design, data analysis, and interpretation of results. We thank Bullet Edits Limited for the linguistic editing and proofreading of the manuscript.

## Author contributions

**Conceptualization:** Huanhuan Lin, Shijian Wang.

**Data curation:** Huanhuan Lin.

**Formal analysis:** Huanhuan Lin.

**Methodology:** Huanhuan Lin.

**Software:** Huanhuan Lin.

**Supervision:** Shijian Wang.

**Validation:** Shijian Wang.

**Visualization:** Huanhuan Lin.

**Writing – original draft:** Huanhuan Lin.

**Writing – review & editing:** Shijian Wang.








