# Supplementary material for: Heavy metal exposures and constipation among United States adults: A cross-sectional study
Source: Medicine (Baltimore). 2026 Oct 9;105(41):e51070. doi: 10.1097/MD.0000000000051070 (PMC13657621; doi:10.1097/MD.0000000000051070)
Supplement: Supplementary file 1 [file medi-105-e51070-s001.docx]

Supplemental Digital Content 1. Full survey-weighted logistic regression analyses of blood heavy metals and constipation

| Exposure | Model 1 OR (95% CI) | P | Model 2 OR (95% CI) | P | Model 3 OR (95% CI) | P |
| --- | --- | --- | --- | --- | --- | --- |
| **Continuous (log-transformed)** |  |  |  |  |  |  |
| Lead | 0.272 (0.201–0.367) | <.001 | 0.502 (0.353–0.714) | <.001 | 0.496 (0.331–0.745) | .004 |
| Cadmium | 1.624 (1.326–1.990) | <.001 | 1.016 (0.822–1.257) | .88 | 1.009 (0.759–1.343) | .94 |
| Mercury | 0.561 (0.457–0.690) | <.001 | 0.625 (0.501–0.780) | <.001 | 0.701 (0.542–0.905) | .01 |
| **Quartiles** |  |  |  |  |  |  |
| Lead Q1 | Reference |  | – | – | Reference |  |
| Lead Q2 | 0.614 (0.505-0.747) | <.001 | 0.749 (0.608-0.923) | .009 | 0.759 (0.546–1.057) | .08 |
| Lead Q3 | 0.455 (0.361–0.573) | <.001 | 0.623 (0.479–0.810) | .001 | 0.642 (0.419–0.985) | .05 |
| Lead Q4 | 0.457 (0.349–0.599) | <.001 | 0.687 (0.501–0.941) | .02 | 0.690 (0.417–1.143) | .10 |
| Cadmium Q1 | Reference |  | – | – | Reference |  |
| Cadmium Q2 | 0.923 (0.742–1.148) | .46 | 0.754 (0.613–0.929) | .01 | 0.763 (0.544–1.071) | .09 |
| Cadmium Q3 | 1.155 (0.886–1.506) | .28 | 0.809 (0.629–1.040) | .09 | 0.822 (0.554–1.221) | .21 |
| Cadmium Q4 | 1.395 (1.098–1.773) | .008 | 0.911 (0.713–1.165) | .44 | 0.908 (0.539–1.528) | .60 |
| Mercury Q1 | Reference |  | – | – | Reference |  |
| Mercury Q2 | 0.749 (0.611–0.919) | .007 | 0.757 (0.620–0.925) | .009 | 0.802 (0.579–1.109) | .12 |
| Mercury Q3 | 0.737 (0.577–0.943) | .02 | 0.765 (0.593–0.988) | .04 | 0.835 (0.552–1.262) | .26 |
| Mercury Q4 | 0.521 (0.414–0.655) | <.001 | 0.600 (0.472–0.762) | <.001 | 0.673 (0.463–0.980) | .04 |
