# Supplementary material for: Heavy metal exposures and constipation among United States adults: A cross-sectional study
Source: Medicine (Baltimore). 2026 Oct 9;105(41):e51070. doi: 10.1097/MD.0000000000051070 (PMC13657621; doi:10.1097/MD.0000000000051070)
Supplement: Supplementary file 2 [file medi-105-e51070-s002.docx]

Supplemental Digital Content 2. Detailed subgroup and interaction analyses for the association between blood lead levels and constipation

| **Subgroup** | **OR (95% CI)** | **P value** | **P for interaction** |
| --- | --- | --- | --- |
| Age (years) |  |  | .62 |
| 20–39 | 0.354 (0.192, 0.650) | .002 |  |
| 40–59 | 0.637 (0.340, 1.194) | .15 |  |
| ≥60 | 0.467 (0.205, 1.060) | .07 |  |
| Gender |  |  | .85 |
| Male | 0.374 (0.178, 0.788) | .01 |  |
| Female | 0.533 (0.350, 0.812) | .005 |  |
| Race |  |  | .10 |
| Mexican American | 1.589 (0.766, 3.297) | .20 |  |
| Non-Hispanic White | 0.339 (0.210, 0.547) | <.001 |  |
| Non-Hispanic Black | 0.453 (0.245, 0.837) | .01 |  |
| Other Hispanic | 0.516 (0.199, 1.343) | .16 |  |
| Other Race | 0.767 (0.107, 5.499) | .78 |  |
| Education |  |  | .04 |
| <9th grade | 1.162 (0.627, 2.156) | .62 |  |
| 9–11th grade | 1.211 (0.728, 2.015) | .45 |  |
| High school | 0.409 (0.209, 0.801) | .01 |  |
| Some college | 0.295 (0.121, 0.719) | .009 |  |
| College+ | 0.335 (0.122, 0.921) | .04 |  |
| Marital status |  |  | .87 |
| Married/cohabiting | 0.463 (0.272, 0.789) | .006 |  |
| Unmarried | 0.415 (0.176, 0.981) | .05 |  |
| Separated/Divorced/Widowed | 0.553 (0.226, 1.353) | .19 |  |
| PIR |  |  | .28 |
| ≤1.3 | 0.802 (0.535, 1.202) | .27 |  |
| 1.31–3.49 | 0.397 (0.187, 0.841) | .02 |  |
| ≥3.5 | 0.385 (0.208, 0.710) | .004 |  |
| BMI (kg/m²) |  |  | .27 |
| <25 | 0.375 (0.210, 0.669) | .002 |  |
| 25–29.99 | 0.440 (0.229, 0.848) | .02 |  |
| ≥30 | 0.719 (0.393, 1.314) | .27 |  |
| Smoking status |  |  | .94 |
| Never | 0.524 (0.308, 0.892) | .02 |  |
| Former | 0.479 (0.292, 0.785) | .005 |  |
| Current | 0.441 (0.223, 0.875) | .02 |  |
| Drinking status |  |  | .58 |
| Non-drinker | 0.504 (0.317, 0.801) | .005 |  |
| Mild drinker | 0.301 (0.097, 0.935) | .04 |  |
| Excessive drinker | 0.518 (0.230, 1.165) | .11 |  |
| Moderate activity |  |  | .58 |
| No | 0.438 (0.178, 1.082) | .07 |  |
| Yes | 0.525 (0.318, 0.866) | .02 |  |
| Vigorous activity |  |  | .40 |
| No | 0.442 (0.276, 0.707) | .003 |  |
| Yes | 0.655 (0.296, 1.452) | .26 |  |
| Hypertension |  |  | .64 |
| No | 0.550 (0.324, 0.936) | .03 |  |
| Yes | 0.353 (0.143, 0.877) | .03 |  |
| Diabetes |  |  | .20 |
| No | 0.465 (0.302, 0.716) | .003 |  |
| Yes | 1.042 (0.247, 4.391) | .95 |  |
| eGFR stage |  |  | .24 |
| ≥90 | 0.558 (0.318, 0.980) | .04 |  |
| 60–89 | 0.434 (0.149, 1.269) | .11 |  |
| <60 | 0.133 (0.026, 0.686) | .02 |  |
| Energy intake |  |  | .37 |
| T1 | 0.646 (0.290, 1.441) | .26 |  |
| T2 | 0.257 (0.147, 0.448) | <.001 |  |
| T3 | 0.694 (0.335, 1.440) | .29 |  |
| Fiber density |  |  | .38 |
| T1 | 0.366 (0.192, 0.695) | .005 |  |
| T2 | 0.617 (0.282, 1.351) | .20 |  |
| T3 | 0.562 (0.222, 1.424) | .20 |  |
| Water intake |  |  | .36 |
| T1 | 0.522 (0.278, 0.981) | .05 |  |
| T2 | 0.498 (0.226, 1.100) | .08 |  |
| T3 | 0.447 (0.191, 1.049) | .06 |  |
| Caffeine intake |  |  | .22 |
| T1 | 0.327 (0.156, 0.685) | .007 |  |
| T2 | 0.671 (0.341, 1.318) | .22 |  |
| T3 | 0.610 (0.261, 1.426) | .23 |  |
| Dietary supplements |  |  | .77 |
| No | 0.515 (0.313, 0.846) | .01 |  |
| Yes | 0.493 (0.245, 0.994) | .05 |  |
