# Supplementary material for: Heavy metal exposures and constipation among United States adults: A cross-sectional study
Source: Medicine (Baltimore). 2026 Oct 9;105(41):e51070. doi: 10.1097/MD.0000000000051070 (PMC13657621; doi:10.1097/MD.0000000000051070)
Supplement: Supplementary file 3 [file medi-105-e51070-s003.docx]

Supplemental Digital Content 3. Detailed subgroup and interaction analyses for the association between blood mercury levels and constipation

| **Subgroup** | **OR (95% CI)** | **P value** | **P for interaction** |
| --- | --- | --- | --- |
| Age (years) |  |  | .31 |
| 20–39 | 0.797 (0.546, 1.163) | .23 |  |
| 40–59 | 0.509 (0.339, 0.763) | .002 |  |
| ≥60 | 0.574 (0.357, 0.924) | .02 |  |
| Gender |  |  | .15 |
| Male | 0.835 (0.583, 1.195) | .31 |  |
| Female | 0.564 (0.421, 0.755) | <.001 |  |
| Race |  |  | .18 |
| Mexican American | 0.596 (0.346, 1.026) | .06 |  |
| Non-Hispanic Black | 0.924 (0.645, 1.322) | .65 |  |
| Non-Hispanic White | 0.559 (0.411, 0.759) | <.001 |  |
| Other Hispanic | 0.474 (0.242, 0.929) | .03 |  |
| Other Race | 1.160 (0.481, 2.798) | .73 |  |
| Education |  |  | .90 |
| <9th grade | 0.640 (0.379, 1.082) | .09 |  |
| 9–11th grade | 0.696 (0.379, 1.276) | .23 |  |
| High school | 0.843 (0.526, 1.352) | .47 |  |
| Some college | 0.520 (0.326, 0.829) | .008 |  |
| College+ | 0.539 (0.299, 0.973) | .04 |  |
| Marital status |  |  | .36 |
| Married/cohabiting | 0.633 (0.480, 0.834) | .002 |  |
| Unmarried | 0.860 (0.555, 1.333) | .49 |  |
| Separated/Divorced/Widowed | 0.502 (0.313, 0.807) | .006 |  |
| PIR |  |  | .50 |
| ≤1.3 | 0.593 (0.418, 0.842) | .005 |  |
| 1.31–3.49 | 0.678 (0.457, 1.006) | .05 |  |
| ≥3.5 | 0.625 (0.438, 0.894) | .01 |  |
| BMI (kg/m²) |  |  | .66 |
| <25 | 0.593 (0.422, 0.834) | .004 |  |
| 25–29.99 | 0.735 (0.457, 1.181) | .19 |  |
| ≥30 | 0.560 (0.381, 0.821) | .005 |  |
| Smoking status |  |  | .76 |
| Never | 0.619 (0.477, 0.805) | <.001 |  |
| Former | 0.523 (0.326, 0.837) | .009 |  |
| Current | 0.782 (0.465, 1.317) | .34 |  |
| Drinking status |  |  | .33 |
| Non-drinker | 0.594 (0.449, 0.785) | <.001 |  |
| Mild drinker | 0.893 (0.539, 1.479) | .65 |  |
| Excessive drinker | 0.584 (0.305, 1.119) | .10 |  |
| Moderate activity |  |  | .42 |
| No | 0.599 (0.361, 0.996) | .05 |  |
| Yes | 0.749 (0.562, 0.998) | .05 |  |
| Vigorous activity |  |  | .88 |
| No | 0.730 (0.549, 0.970) | .03 |  |
| Yes | 0.649 (0.413, 1.019) | .06 |  |
| Hypertension |  |  | .92 |
| No | 0.693 (0.512, 0.939) | .02 |  |
| Yes | 0.777 (0.487, 1.239) | .26 |  |
| Diabetes |  |  | .97 |
| No | 0.692 (0.524, 0.916) | .02 |  |
| Yes | 0.722 (0.324, 1.609) | .39 |  |
| eGFR stage |  |  | .38 |
| ≥90 | 0.731 (0.523, 1.022) | .06 |  |
| 60–89 | 0.725 (0.496, 1.060) | .09 |  |
| <60 | 0.351 (0.151, 0.817) | .02 |  |
| Energy intake |  |  | .56 |
| T1 | 0.658 (0.425, 1.018) | .06 |  |
| T2 | 0.742 (0.474, 1.162) | .17 |  |
| T3 | 0.822 (0.483, 1.398) | .43 |  |
| Fiber density |  |  | .73 |
| T1 | 0.644 (0.422, 0.985) | .04 |  |
| T2 | 0.716 (0.456, 1.122) | .13 |  |
| T3 | 0.791 (0.509, 1.230) | .27 |  |
| Water intake |  |  | .34 |
| T1 | 0.663 (0.446, 0.988) | .04 |  |
| T2 | 0.869 (0.554, 1.362) | .51 |  |
| T3 | 0.573 (0.362, 0.908) | .02 |  |
| Caffeine intake |  |  | .12 |
| T1 | 0.745 (0.500, 1.110) | .13 |  |
| T2 | 0.890 (0.580, 1.367) | .56 |  |
| T3 | 0.494 (0.272, 0.894) | .02 |  |
| Dietary supplements |  |  | .41 |
| No | 0.764 (0.518, 1.125) | .15 |  |
| Yes | 0.617 (0.463, 0.824) | .004 |  |
