# Supplementary material for: Heavy metal exposures and constipation among United States adults: A cross-sectional study
Source: Medicine (Baltimore). 2026 Oct 9;105(41):e51070. doi: 10.1097/MD.0000000000051070 (PMC13657621; doi:10.1097/MD.0000000000051070)
Supplement: Supplementary file 4 [file medi-105-e51070-s004.docx]

Supplemental Digital Content 4. Detailed subgroup and interaction analyses for the association between blood cadmium levels and constipation

| **Subgroup** | **OR (95% CI)** | **P value** | **P for interaction** |
| --- | --- | --- | --- |
| Age (years) |  |  | .52 |
| 20–39 | 0.863 (0.515, 1.447) | .56 |  |
| 40–59 | 0.960 (0.491, 1.879) | .90 |  |
| ≥60 | 1.877 (1.118, 3.151) | .02 |  |
| Gender |  |  | .76 |
| Male | 0.953 (0.477, 1.906) | .89 |  |
| Female | 1.092 (0.772, 1.545) | .60 |  |
| Race |  |  | .29 |
| Mexican American | 0.498 (0.254, 0.977) | .04 |  |
| Non-Hispanic White | 1.131 (0.735, 1.741) | .56 |  |
| Non-Hispanic Black | 0.996 (0.598, 1.660) | .99 |  |
| Other Hispanic | 0.918 (0.284, 2.967) | .88 |  |
| Other Race | 1.439 (0.215, 9.654) | .70 |  |
| Education |  |  | .23 |
| <9th grade | 1.579 (0.562, 4.437) | .37 |  |
| 9–11th grade | 0.797 (0.334, 1.902) | .60 |  |
| High school | 0.648 (0.345, 1.217) | .17 |  |
| Some college | 1.439 (0.800, 2.585) | .21 |  |
| College+ | 1.580 (0.493, 5.059) | .43 |  |
| Marital status |  |  | .79 |
| Married/cohabiting | 1.119 (0.703, 1.782) | .62 |  |
| Unmarried | 0.732 (0.368, 1.457) | .36 |  |
| Separated/Divorced/Widowed | 1.034 (0.464, 2.308) | .93 |  |
| PIR |  |  | .87 |
| ≤1.3 | 0.803 (0.463, 1.392) | .42 |  |
| 1.31–3.49 | 0.988 (0.609, 1.603) | .96 |  |
| ≥3.5 | 1.432 (0.596, 3.442) | .41 |  |
| BMI (kg/m²) |  |  | .64 |
| <25 | 1.308 (0.717, 2.388) | .37 |  |
| 25–29.99 | 1.015 (0.614, 1.676) | .95 |  |
| ≥30 | 0.719 (0.428, 1.209) | .20 |  |
| Smoking status |  |  | .37 |
| Never | 0.705 (0.428, 1.161) | .16 |  |
| Former | 2.112 (0.951, 4.688) | .07 |  |
| Current | 1.156 (0.682, 1.959) | .58 |  |
| Drinking status |  |  | .36 |
| Non-drinker | 1.147 (0.845, 1.556) | .36 |  |
| Mild drinker | 1.053 (0.355, 3.126) | .92 |  |
| Excessive drinker | 0.536 (0.205, 1.406) | .20 |  |
| Moderate activity |  |  | .89 |
| No | 0.901 (0.456, 1.781) | .74 |  |
| Yes | 1.072 (0.686, 1.676) | .74 |  |
| Vigorous activity |  |  | .63 |
| No | 1.008 (0.736, 1.379) | .96 |  |
| Yes | 0.983 (0.530, 1.825) | .95 |  |
| Hypertension |  |  | .71 |
| No | 1.059 (0.722, 1.554) | .75 |  |
| Yes | 0.899 (0.478, 1.692) | .72 |  |
| Diabetes |  |  | .51 |
| No | 1.023 (0.750, 1.395) | .87 |  |
| Yes | 0.861 (0.312, 2.379) | .75 |  |
| eGFR stage |  |  | .59 |
| ≥90 | 0.936 (0.656, 1.333) | .68 |  |
| 60–89 | 1.161 (0.509, 2.651) | .70 |  |
| <60 | 1.453 (0.393, 5.365) | .53 |  |
| Energy intake |  |  | .82 |
| T1 | 0.747 (0.424, 1.315) | .28 |  |
| T2 | 1.303 (0.628, 2.707) | .44 |  |
| T3 | 1.128 (0.522, 2.435) | .74 |  |
| Fiber density |  |  | .91 |
| T1 | 1.085 (0.659, 1.787) | .73 |  |
| T2 | 0.882 (0.452, 1.718) | .69 |  |
| T3 | 1.134 (0.479, 2.681) | .75 |  |
| Water intake |  |  | .09 |
| T1 | 1.256 (0.773, 2.039) | .32 |  |
| T2 | 0.683 (0.374, 1.247) | .19 |  |
| T3 | 1.009 (0.537, 1.895) | .98 |  |
| Caffeine intake |  |  | .65 |
| T1 | 1.266 (0.720, 2.226) | .38 |  |
| T2 | 0.986 (0.567, 1.716) | .96 |  |
| T3 | 0.808 (0.419, 1.558) | .49 |  |
| Dietary supplements |  |  | .13 |
| No | 1.330 (0.892, 1.983) | .14 |  |
| Yes | 0.668 (0.367, 1.215) | .16 |  |
